# Diseases of the Teeth and Their Relation to the General Health

**Published:** 1891-02

**Authors:** W. M. Williams

**Affiliations:** Cincinnati


### Diseases of the Teeth and Their Relation to the General Health.

BY W. M. WILLIAMS, M.D., CINCINNATI.

A paper read before the Cincinnati Medical Society, November 11, 1890.

Diseases of the teeth have become so common that it is a subject of paramount importance—a subject of importance to the physician, as well as to the dental specialist, and I shall indeavor in this paper to bring to your attention what on the subject should be of special interest to the general practitioner.

Caries of the teeth, which is the most common affection, is largely a disease of childhood. The teeth erupt in an imperfect state of development as to structure. The ossification is incomplete; the center of the tooth is largely pulp, which each year becomes smaller, until in old age the pulp is almost obliterated. The dentine, under favorable circumstances, becomes harder with age. Hence, we see that in childhood the teeth are less able to resist destructive agents, and decay does not have to penetrate far to reach the pulp.

Other causes than age predispose the teeth to decay. Sex is to be considered. Women do not have as good teeth as men for the reason that their health is less rugged. Any wasting disease, such as fevers or consumption, has its effect on the teeth, particularly in a person of thirty or thirty-five years.

Pregnancy has a most marked effect. Women whose teeth have always been good, often experience much trouble from caries after having had children. Indeed, this is quite the rule. Heredity is always a constant factor.

Caries of the teeth having once begun rarely ceases to be progressive, and unless checked by treatment will go on to the destruction of the tooth, and it is most likely to involve adjacent teeth. After the caries has penetrated to the pulp a more serious condition exists. Especially is this so in children, and it is apt to involve the loss of the tooth. On the exposure of the pulp much pain ensues, which continues usually until the pulp dies, after which the tooth may remain quiet for a time, until the pulp undergoes decomposition, and the products of the decomposition escaping through the foramen at the apex of the root into the tissues beyond, set up an acute inflammation which usually runs a definite course, and after much suffering, and often great swelling of the face, terminates in an alveolar abscess at a point near the apex of the root of the tooth involved, and if the tooth be not removed or thoroughly treated this abscess will remain open and become chronic.

This condition may terminate in other ways. If the diseased tooth be a superior first molar the abscess may break into the antrum of Highmore, and cause an abscess of that cavity. Fortunately this is not a very common complication, but I have seen a number of cases of it. These abscesses occasionally cause necrosis of the jaw bone, and another serious result which frequently occurs is where one of the inferior first permanent molars of a child from eight to fifteen years old becomes abscessed. The tissues being soft and yielding, the pus burrows and gravitates and forms an external opening, usually under the jaw. Sometimes there are several of these openings, forming unsightly scars. The salivary and lymphatic glands in the neighborhood generally become infiltrated. Such results are likely to happen in any case, but the sure preventive is the extraction of the tooth.

There are cases reported where a general blood poisoning has resulted from abscessed teeth.

Neuralgia is commonly associated with diseases of the teeth. In people of the neuralgic tendency one of the branches of the fifth pair of nerves may be the seat of the most severe pain having its origin in dental lesions, and causes of the most cruel and persistent facial neuralgia are often entirely cured by the removal or treatment of diseased teeth.

While these are the most serious troubles that result from dental lesions, there are other consequences that invariably attend them, dependent on the extent of the caries, or number of teeth involved or already lost. Mastication can not be perfectly performed owing to loss of teeth, or pain in the diseased ones, and thereby food is taken into the stomach but imperfectly prepared for it, and as a consequence there are alimentary disturbances and mal-nutrition. I have known people to increase rapidly in weight after having had their teeth restored to a healthy condition or artificial ones inserted. Great as the effect of diseased teeth is, in an ordinarily healthy person, much more would a person who is sick from other causes suffer therefrom, and convalescence may be greatly retarded.

A very offensive breath is caused by unhealthy conditions of the oral cavity, not alone always due to carious teeth, but to inflammation of the gums; also caused by salivary calculi or tartar.

It is common in people who have neglected their teeth to find nearly half of the thirty-two teeth largely broken down, and the gums tumefied from diseased roots. Nor is this condition found only in the ignorant and poorer classes. People who have intelligence and means neglect themselves through want of thought or information on the subject, only to regret it bitterly when too late It is the duty of a physician, who is the custodian of the health of his patients, to advise them on this subject, and to see that their health and comfort does not suffer by this neglect.

DISCUSSION.

Dr. C. R. Holmes: A prominent young lady of the city came to me, a short time ago, on account of intense pain in the left ear, and radiating down the neck. She attributed it to a nasal catarrh which she had. Treatment availed nothing. I sent her away for a change of climate. She returned home with the same old story of pain in the ear. I examined the Eustachian tube, but could find nothing. Finally one day I noticed that the gum was swollen just where the wisdom tooth was coming through. I introduced a probe and got pus. I then sent her to a dentist and the tooth was extracted. Shortly afterwards she came to my office again. She had swelling along the back of the neck extending down to the scapula. I gave her a placebo and all the symptoms disappeared the next day.

Dr. C. E. Caldwell: Reflex neuralgia due to the teeth is common. I have had a number of such cases. Whenever a case of severe ear-ache comes to my office I always first inspect the teeth, and often find here the cause of the neuralgia. One case of severe facial neuralgia I sent to a dentist. He sent her back with the statement that the teeth were all good, and that the neuralgia was due to malaria. I afterwards found a small abscess at the root of one of the teeth, which I opened, and the neuralgia then disappeared.

Dr. Fitzpatrick: I would like to ask the essayist how he would differentiate between neuralgia due to the teeth and neuralgia due to the presence of intra-nasal hypertrophies.

Dr. Williams: The way to differentiate would be to examine the teeth, and if they are all sound look elsewhere.

Dr. Thompson: A probable answer to Dr. Fitzpatrick's question could be made by the application of a solution of cocaine to the nose. The same is also true of the teeth. By the application of cocaine here you can often allay the neuralgia.

Dr. Thorner: Does not facial neuralgia sometimes result from the teeth even when there is no caries.

Dr. Williams: A tooth that has a live pulp can not have an abscess. By sounding the tooth, or drilling into it, you can ascertain whether the pulp is dead or alive.

---

Deductions from Experiments with Drugs.—The *Progres Medical* states that Dr. Huchard recently read a paper on "The Physiological and Therapeutical Action of Drugs" before the *Societe de Therapeutique*, calling attention anew to significant differences in the action of certain drugs in the well and in the sick, and in various forms of disease. For example, it was stated that quinine lowered the temperature in typhoid fever, but had no such effect in erysipelas. The lesson to be drawn from such facts is that it is not safe to make sweeping therapeutic deductions from observations of the physiological action of drugs. To use the author's words, "Physiology should not enslave medicine."—*N. Y. Med. Journal.*

---

As a local anæsthetic Dr. J. B. Mattison recommends a spray of the following compound, viz.: Menthöl one drachm, chloroform ten drachms, ether fifteen drachms.

